# Evaluation of Bax and Bcl-2 Proteins Expression in the Rat Hippocampus due to Childhood Febrile Seizure

**Published:** 2016

**Authors:** Mohammad Javad SAEEDI BORUJENI, Javad HAMI, Hossein HAGHIR, Maryam RASTIN, Ghasem SAZEGAR

**Affiliations:** 1 Department of Anatomy and Molecular Biology, School of Medicine, Isfahan University of Medical Sciences, Isfahan, Iran; 2 Isfahan Neurosciences Research Center, Al-Zahra Hospital, Isfahan University of Medical Sciences, Isfahan, Iran; 3 Department of Anatomical Sciences, School of Medicine, Birjand University of Medical Sciences, Birjand, Iran; 4 Department of Anatomy and Cell Biology, School of Medicine, Mashhad University of Medical Sciences, Mashhad, Iran; 5 Medical Genetic Research Center (MGRC), Mashhad University of Medical Sciences, Mashhad, Iran; 6 Immunology Research Center , Bu-Ali Research Institute, Mashhad University of Medical Sciences, Mashhad, Iran

**Keywords:** Simple Febrile Seizure, Hippocampus, Apoptosis

## Abstract

**Objective:**

Simple Febrile Seizure (SFS) is the most common seizure disorder in childhood, and is frequently described as inoffensive disorder. Nevertheless, there is evidence suggesting the association between neonatal febrile seizures and hippocampal abnormalities in adulthood. This study was conducted at evaluating the hippocampal expression of pro-apoptotic Bax and anti-apoptotic Bcl-2 proteins following SFS induction in rat neonates.

**Materials & Methods:**

Febrile seizure was modeled by hyperthermia-induced seizure in 22-dayold male rats by a hot water bath. The animals were divided into two groups based on the presence or absence of seizure behaviors: Hyperthermia without seizure (n=10) and hyperthermia with seizure (n=10). To control the effects of environmental stress a sham-control group was also added (n=10). The rats’ hippocampi were dissected 2 or 15 days after hyperthermia. The expression of Bax and Bcl-2 proteins were measured using Western Blotting technique.

**Results:**

The hippocampal expression of Bcl-2 protein was significantly lower in the hyperthermia with seizure animals than that of the sham-control and hyperthermia without seizure groups. The expression of pro-apoptotic Bax protein also significantly increased in the hippocampus of hyperthermia with seizure group rats compared to the sham-control and hyperthermia without seizure animals.

**Conclusion:**

The simple febrile seizure markedly disturbed the hippocampal expression of both Bcl2 and Bax proteins, resulting in apoptosis promotion in hippocampi of juvenile rats, which were measurable for at least 15 days.

## Introduction

Simple Febrile Seizures (SFS) are the most common type of seizure in children, and occur in 2-5% of young children (1), although the etiology of this kind of seizures remains unknown (2). A febrile seizures is a full-body convulsion in infants or small children triggered by high fevers (3). In humans, the fever-induced seizures are mainly seen in children aged between 6 months to 5 years; and classified into two major types as SFS and complex febrile seizure (CFS) (1-3). These types of seizures in children occur suddenly. However, following acute febrile illness, the assessment of the various aspects of seizure is not possible (3). In addition, the parent’s reports have not a high degree of scientific validity. On the other hand, answer to many key questions including the mechanisms of these types of seizures and acute and chronic effects on brain are not possible in human studies (4). Therefore, clinical and basic scientists attempt to create a simulation with different models for experimental febrile convulsions in the lab. Because seizure behaviors are directly observable and recordable in animals, and it is possible to study the animal’s brain after seizure, the experimenting on animal is acceptable and frequently used to assess the impacts and mechanisms of FS (4). Hippocampus is a part of limbic system located in the inner part of temporal lobe cortex and its role has well been documented in learning and memory (5). CFS has been associated statistically with the development of intractable epilepsy that involves the limbic circuit (6, 7). However, according to following studies, SFS is benign and is associated with hippocampal anomalies in adults. A recent cross-sectional MRI survey in healthy adults who experienced SFS in childhood showed that the SFS could cause persistent hippocampal abnormalities even after more than 15 years (8). A positive correlation between SFS with sudden unexpected death in human kids is reported recently (9). Apoptosis is a genetically controlled form of cell death that occurs in wide variety of physiological and pathological processes (10, 11). Morphologically, apoptosis is characterized by a series of structural changes such as swelling of the cell membrane, cell shrink, nuclear condensation and DNA fragmentation (12). Bcl-2 family proteins are one of the key regulators of apoptosis (13, 14). At least, 15 Bcl-2 family members have been identified so far in mammalian cells (15). They function as either pro-apoptotic Bax or anti-apoptotic Bcl-2regulators) (13, 14). The ratio of anti- and proapoptotic proteins determines in part how cells respond to apoptotic or survival signals. Because of current controversies concerning the probable effects of SFS on hippocampal cell apoptosis, molecular and basic studies surviving possible pathological effects of SFS on the hippocampi of juvenile rats can resolve many of these contradictions. Therefore, this study aimed to analyze the expression of pro-apoptotic Bax and anti-apoptotic Bcl-2 proteins in the hippocampus of rats triggered by neonatal simple febrile seizure.

## Materials& Methods

**Study Design and Experimental Groups**

Thirty adult Wistar female rats (200-250 g body weight, 6-8 wk old) were purchased from Mashhad University of Medical Sciences Experimental Animal House (Mashhad, Iran). Animals were housed in individual cages at 22 ± 2 °C with free access to pellet food and water and on a 12 h light/dark cycle. They were fed a regular rat chow. Female animals were mated with males of the same strain. At the end of pregnancy, the animals were allowed to deliver naturally. The day of birth was defined as postnatal day 1 (P1).Before starting he experiments, body weights and rectal body temperatures of pups were measured on day 22 after birth. To induce hyperthermia, we used water bath model. Therefore, the 22-day-old male rats were placed inside of one tiled water tank (30 × 30 × 30 cm) for 4 min. Water temperature was 45 °C (16, 17). The animals were divided into two groups based on the presence or absence of the hyperthermia-induced behavioral seizures as follows: Hyperthermia with seizure group (n=10) Hyperthermia without seizure group (n=10) To assess the intensity of seizure, we used a four-stage scale: 1) no convulsions, 2) facial clonus and/or head nodding, 3) forelimb clonus and 4) rising with laterally extended back limbs and occasionally monitored by falling back (18). Seizure duration was recorded by a digital stopwatch. After hyperthermia at 45 °C for 4 min, ten animals that experienced 4th seizure’s stage were randomly selected (as hyperthermia with seizure group). To control the effects of environmental stress a sham-control group consisting 10 pups also included in the study. The sham- control animals were placed in a water bath at 37 °C for 4 min. In order to determine the probable permanence of molecular changes due to hyperthermia or seizure, the animals then were subdivided into two groups of five members as follows: Group A: The pups were scarified after 2 days post experiment. Group B: The animals were killed at 14 days after experiment. Rats were deeply anesthetized with chloral hydrate (350 mg/kg). The whole brain was rapidly removed and placed on an ice-cooled cutting board. The meninges were carefully removed and hippocampus was dissected from hemispheres, snap frozen in liquid nitrogen and stored at −70°C for extraction of protein.

**Ethics**

The study protocol followed the ethical principle outlined by the Helsinki Declaration and was approved by the Ethics Committee of the School of Medicine, Mashhad University of Medical Sciences, Mashhad, Iran.

**Western blotting**

The expression of Bax and Bcl-2 proteins in animal hippocampi were measured by western blotting. The hippocampal tissues collected were homogenized with ice-cold homogenizing buffer (50mMTris-Hcl, 150 mMNaCl, 1mM EDTA, 0.5mM Triton X100, pH 7.4) containing protease inhibitor cocktail tablet (Roche, Germany). We used Bio-Rad protein assay for determining protein concentration. Hippocampal protein lysates were exposed to SDS-PAGE (12.5%) under reducing conditions and transferred to a polyvinylidenedifluoride (PVDF) membrane (Millipore, USA). Membranes were treated with Attoglow western blot system kit, according to the manufacturer protocol (Biochain, USA). Briefly, blots were blocked with blocking buffer (5% skimmed milk powder in PBS) for 2 h. After blocking, blots were incubated with rabbit anti-Bcl-2 polyclonal antibody (1/500, v/v), rabbit anti-Bax polyclonal antibody (1/250, v/v) (Biovision, USA), and rabbit anti-B-Actin polyclonal antibody (1/1000, v/v) (SIGMA-ALDRICH, USA) for 20 h at 4 °C. Blots were washed for 4 times with 0.1% tween 20 in PBS and incubated with HRP conjugated-secondary antibody (1/5000, v/v, Biochain, USA) for 1 h at room temperature. The Bcl-2 and Bax protein bands were visualized using enhanced chemiluminescnces (ECL) method (Bioimaging, system, syngene, UK) and quantified using ImageJ software.

**Statistical Analysis**

Statistical analysis was performed using the SPSS statistical package, version 15.0 (Chicago, IL, USA). Significant differences (P< 0.05) between groups were determined using an independent sample t test and one-way analysis of variance (ANOVA) followed by Tukey’s test. A P value of less than 0.05 was accepted as statistically significant.

## Results

**Body weight and Body temperature**

Before applying hyperthermia, the mean body weight and body temperature of rat pups on postnatal day 22 were 46 ± 0.3 gr and 36.7±0.2 °C, respectively. Our data showed that hot water bath model significantly increased the mean body temperature in animals of both studied groups compared to sham – control group (P<0.05, and P<0.01 respectively, Table 1).

**Expression of hippocampal Bax and Bcl-2 proteins**

**Fig. 1:**
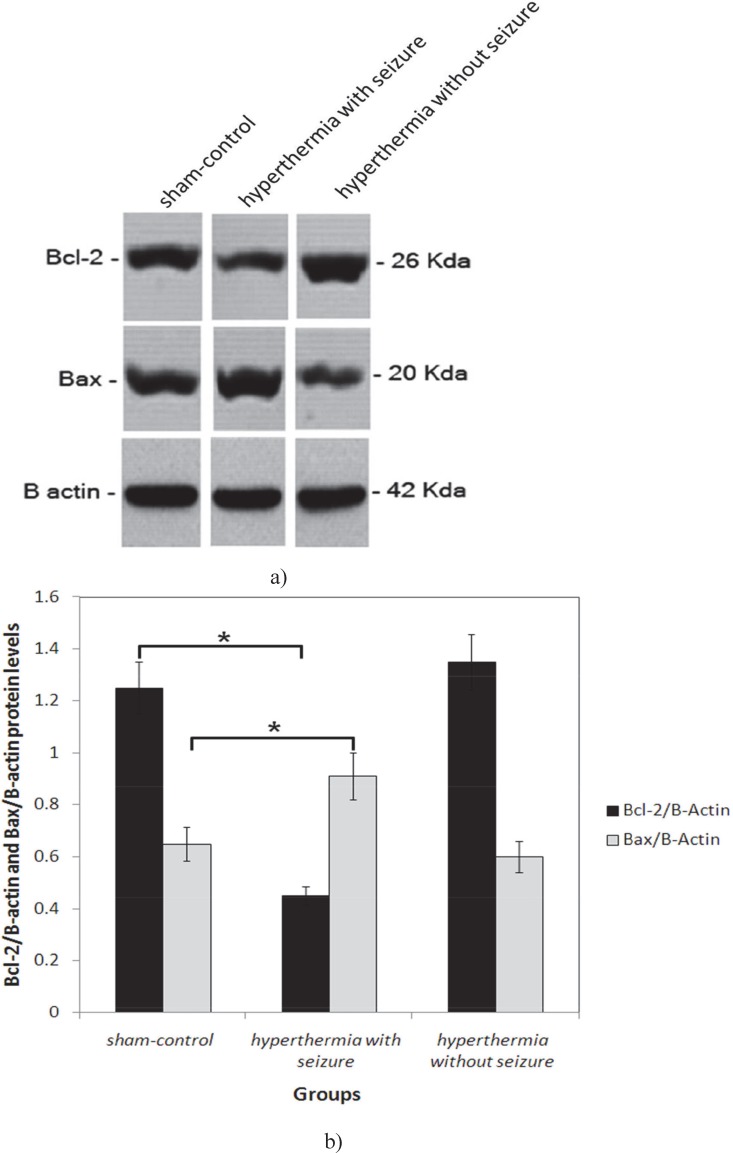
Bax and Bcl-2 protein levels in newborn hippocampi were analyzed by Western blot analysis two days after hyperthermia induction (a). The protein bands were quantified using imageJ analysis software and normalized to β-actin expression (b). Data are presented as means ± S.E.M., n=5–7.

**Fig.2:**
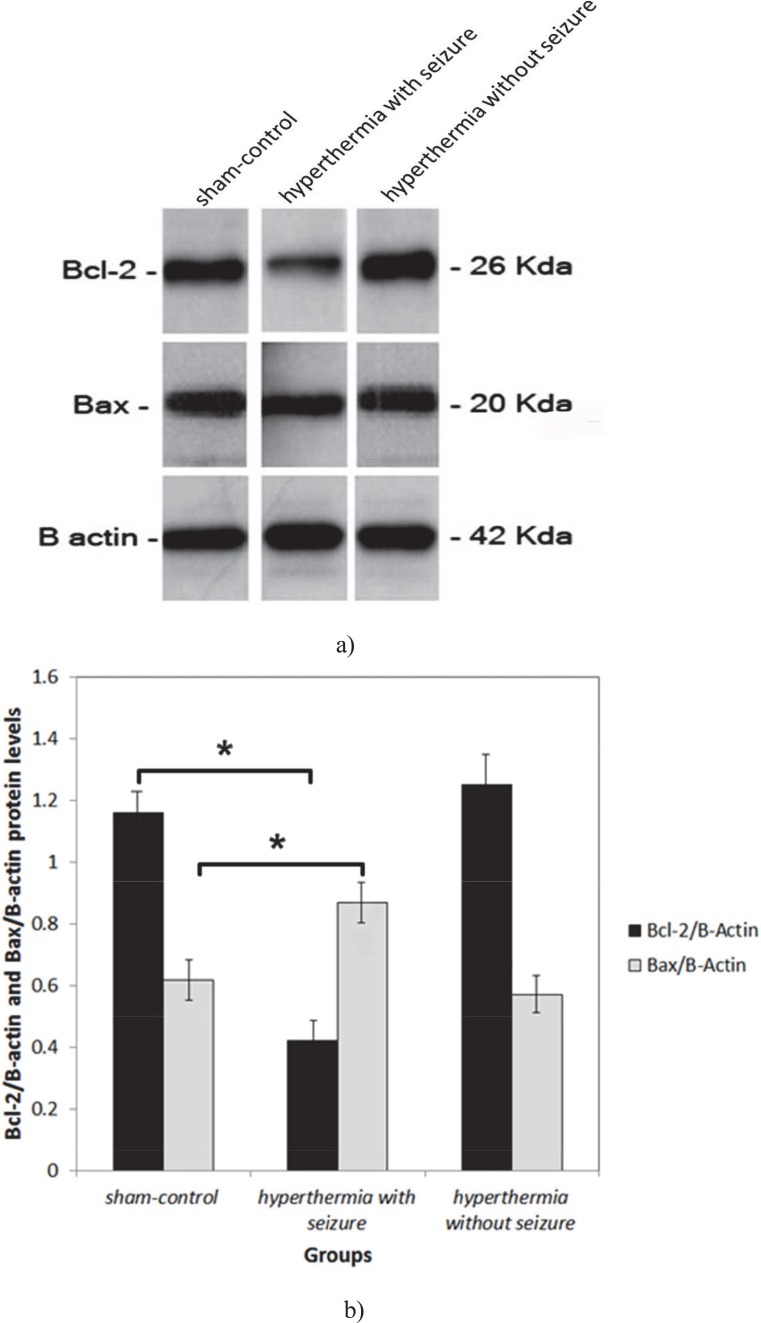
Bax and Bcl-2 protein levels in newborn hippocampi were analyzed by Western blot analysis two weeks after hyperthermia induction (a). The protein bands were quantified using imageJ analysis software and normalized to β-actin expression (b). Data are presented as means ± S.E.M., n=5–7.

Two days after hyperthermia induction, the expression of Bax protein were increased significantly in hippocampi of hyperthermia with seizure group pups compared with those of the hyperthermia without seizure and in the sham-control groups (P0.05 each, Fig.1a, b). Two weeks after experiments, we found a significant increase in expression of Bax protein in hippocampus of hyperthermia with seizure group animals in comparison to hyperthermia without seizure and sham-control rats (P<0.001, and P<0.05 respectively, Fig.2a,b). Nevertheless, the hippocampal expression of Bcl-2 protein was significantly down regulated in hyperthermia with seizure group compared to those of the hyperthermia without seizure and sham-control groups (P<0.01, each, Fig.2a, b). We found no significant difference between the expression of Bax and Bcl-2 proteins in hippocampus of hyperthermia without seizure with sham-control animals (P≥0.05; Fig.2a, b)

## Discussion

A number of different models have been proposed for the simulation of febrile seizures. In the present study, hot water bath model was used to induce febrile seizures, a method with highly controlled condition frequently used to induce febrile seizures (19). Using hot water bath model could successfully increase the mean body temperature in hyperthermia- exposed animals compared to sham-control group in our study. Interestingly, the mean body temperature of the animals in hyperthermia without seizure group also indicated a significantly increase compared to the sham -control group. However, there are no known reasons to explain the exact cause for presence or absence of seizures after induction of hyperthermia. One of the most important points, which must be considered in selecting an appropriate animal model is the age of animals, to reflect the same age in human (4), because the brain regions are different in terms of growth, neurogenesis, migration and function in various ages. On the other hand, the absolute equivalence in age between rat and human brain is not clear. Nonetheless, several studies have been conducted to compare the human and rat brain. Accordingly, a 5 to 7-day-old rat brain is equivalent only to a human brain at term (20); the 15-days-old rat brain is equivalent to a human brain a few months to 1 yr old, and the 28 to 30 days old rat to a 2 yr old child (21).Based on the existence of anatomical and developmental differences between rat and human brains and because of the risk of temporal lobe epilepsy in patients who experienced febrile seizuresbetween the ages of 6 months to 5 years (21), we decided to do this study on 22- day- old rats brain(17, 18). In rat neonates, hyperthermia causes various stereotypical behaviors, including body tonic flexion with facial clonus and demonstrates severe disturbances in brain limbic regions(22). Increased temperature alone plays an important role in the development of febrile seizures(23). Changes in neuronal properties and synaptic connections might play a role in the mechanism of febrile seizures (24). Different models have shown that the intrinsic properties of neurons and synaptic potentials are common temperature-dependent events (24). We showed that hyperthermia alone could not cause an intense increase in apoptosis in the hippocampus of neonatal rats, while hyperthermia with seizure was associated with increased expression of Bax and decreased expression of Bcl-2 proteins in animals’ hippocampi, in consistent with the results of Toth et al. (25) that reported a severe neuronal damage following acute hyper thermic seizures. Their results indicated that the CA1 and CA2 regions of the hippocampus were damaged due to acute hyper thermic seizureand most of these damages were observed in pyramidal and granular cells (25). Our results also are in agreement with the results of Nazem et al. (18). They investigated the effects of simple febrile seizures on neuronal injury and cytogenesis after simple febrile seizure in the hippocampal dentate gyrus of juvenile rats using histological techniques. Accordingly, dark neurons in the dentate gyrus of hippocampus of neonatal rats following SFS were observed for at least 2 weeks (18). In our study, also neuronal apoptosis was measured for at least two weeks. In another study, the researchers investigated the influences of hyperthermia – induced seizures on learning and memory of rats (26).The hyper thermic seizures were induced on postnatal days 10 to 12 and the memory and learning of rats were examined at adolescence and adulthood using Morris water maze test. Their study showed early-life brief but recurrent hyper thermic seizures caused long-term cognitive impairment. In the present study, remarkable alterations in expression of genes associated with promotion of hippocampal apoptosis were seen. We did not examin memory and learning functions, but it seems that memory deficiency is seen in rats’ pups for at least 15 days. Further studies are necessary to clarify the correlation between febrile seizure induced hippocampal apoptosis and behavioral deficiencies. Cassim et al. study also showed exposure to early febrile convulsion on postnatal day 14 might impair cognitive behavioral function (27).Hippocampal injury such as hippocampal edema and later mesial temporal sclerosis could sometimes occur during prolonged and focal febrile seizures in infants who otherwise seem normal (28). Such lesions may raise the risk of focal and prolonged seizures, which in turn may result in more hippocampal injuries (28). Results of present study also are in agreement with the results of Kinney et al. (9). Their survey that performed on children with sudden death with positive history of febrile seizure showed minor pathologic findings in the hippocampus of some patients (9).

**Table 1:** Comparison of The Animal’s Body Temperature in Different Group Studied

**Group**	**Body temperature (°C)**
**sham – control**	**36.5±0.7**
**Hyperthermia with seizure**	**41.5±0.98** **
**Hyperthermia without seizure**	**38.5±0.81** *

* *P*<0.05 versus sham-control group

** *P*<0.01 versus sham-control group

*: Significant Differences (*P*≤0.05)

*: Significant Differences (*P*≤0.05)

**In conclusion**, SFS causes remarkable changes in the expression of both Bcl2 and Bax proteins, and promotes cellular apoptosis in hippocampi of juvenile rats. The present data may prompt clinicians to perform cautious cognitive and memory tests on children with positive history of SFS.

## Notes

**Authors’ contribution** Mohammad JavadSaeediBorujeni: Writing the article Javad Hami: Edition of paper, Statistical analysis

Hossein Haghir: Study planning

Maryam Rastin: Supervision in molecular surveys

Ghasem Sazegar: Scientific supervision, Corresponding author
